# A novel *TPD52L2-ROS1* gene fusion expanding the molecular alterations in inflammatory myofibroblastic tumor: case report and literature review

**DOI:** 10.1186/s13000-023-01382-0

**Published:** 2023-09-21

**Authors:** Xuguang Liu, Yaqi Duan, Guoping Wang, Pengcheng Zhu

**Affiliations:** 1 Department of Pathology, Tongji Hospital, Tongji Medical College, Huazhong University of Science and Technology, Wuhan, 430030 China; 2 Department of Pathology, School of Basic Medical Science, Tongji Medical College, Huazhong University of Science and Technology, No. 13 Hangkong Road, Wuhan, 430030 China

**Keywords:** IMT, *ROS1*, *TPD52L*2, Case report, Crizotinib

## Abstract

**Background:**

Inflammatory myofibroblastic tumor (IMT) is a distinctive tumor composed of spindle cells accompanied by mixed inflammatory cells, and immunohistochemical positivity for ALK (anaplastic lymphoma kinase protein) can be detected in half of IMTs. The diagnosis of ALK-negative IMT could be a challenge. Recently, the fusions of some kinase genes, such as *RET*, *NTRK1*, *ROS1*, etc., are revealed in ALK-negative IMT.

**Case presentation:**

A 19-year-old woman presented with swelling of the left upper arm. Magnetic resonance imaging (MRI) scan revealed a tumor in the left postbrachium extended to the left axillary, serratus anterior muscle, and latissimus dorsi muscle. Histopathologically, the irregular-circumscribed tumor was composed of dense spindle-shaped cells with eosinophilic abundant cytoplasm and hyalinized mesenchyme in an inflammatory background. Immunohistochemically (IHC), tumor cells were positive for SMA, MDM2, and p16; the cells were negative for desmin, MyoD1, Myogenin, pan-cytokeratin, S100, SOX10, HMB45, Malen-A, CD34, CD31, CD99, and ALK. By RNA-based NGS, a novel fusion between TPD52L2 3’ end of exon 1–4 and ROS1 5’ end of exon 36–43 was revealed. ROS1 IHC staining was negative. The final diagnosis of IMT with TPD52L2-ROS1 fusion was made. Subsequently, the patient experienced a good clinical response to Crizotinib, and clinical follow-up showed stable disease after 9 months.

**Conclusion:**

This report expands the spectrum of *ROS1* gene rearrangements in the IMT and highlights the importance of molecular analysis of IMT for getting a diagnostic clue and determining potential therapeutic strategies.

## Introduction

Inflammatory myofibroblastic tumor (IMT) is a distinctive fibroblastic/myofibroblastic neoplasm with chronic inflammatory cells infiltration. IMT affects mainly children and young adults, and the most frequent anatomic sites are the lung, mesentery, omentum, and other anatomic locations including extremities.

More than 50% of IMTs contain rearrangements of the anaplastic lymphoma kinase (ALK) gene on chromosome 2p23, resulting in the aberrant expression of ALK chimeric protein immunohistochemically [1, 2]. Hence, ALK inhibitors have emerged as a successful therapy for ALK-associated IMT in both adult and pediatric patients [3]. Molecular analysis has helped distinguish IMT as a distinctive neoplasm largely characterized by receptor tyrosine kinase (RTK) activation from other mimics.

In addition to the recurrent *ALK* arrangement, ALK-negative IMTs have recently revealed gene rearrangements related to other kinase genes by next-generation sequencing (NGS), such as *NTRK3*, *RET*, *ROS1*, and *PDGFRB* [4–7]. *ROS1* rearrangements have been identified in about 10% of IMT [5, 6, 8]. Surgical excision is the mainstay of treatment for IMT, and targeted molecular therapy could be an effective supplementary.

In this article, we report a case of IMT with typical histopathologic features and a previously undescribed gene fusion involving the *TPD52L2* and *ROS1* gene in a young adult, which expanded the spectrum of IMTs with the novel molecular findings. Furthermore, this patient experienced a good clinical response to Crizotinib, which might indicate that this novel gene fusion results in oncogenic activation in IMT.

## Case presentation

**Fig. 1:**
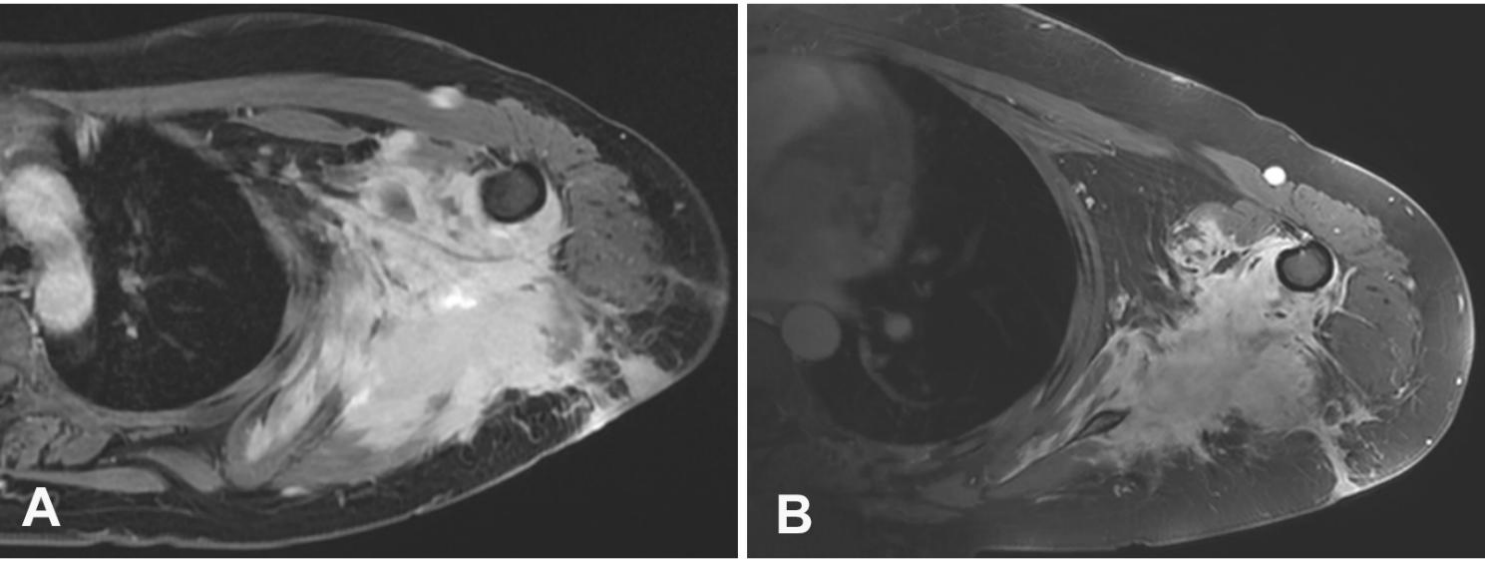
(**A**) Solid 147-mm mass in the left upper arm (MRI scan) before the treatment. (**B**) After one month of TKI treatment, the max size of mass was reduced to 108 mm (MRI scan)

A 19-year-old woman presented with a chief complaint of swelling and pain in the left upper arm that had been growing slowly over the previous 1 year. Physical examination showed thickened left upper arm and shoulder, which, on magnetic resonance imaging (MRI) scan was identified as a mass lesion (147 × 116.8 mm) occupying the soft tissue of postbrachium extended to the left axillary, serratus anterior muscle, and latissimus dorsi muscle. (Fig. 1A) During the operation, a mass with irregular boundary, adhered to the blood vessel and nerves, was exposed, which could not be resected completely, subsequently, the partial tumor resection was performed.

**Fig. 2:**
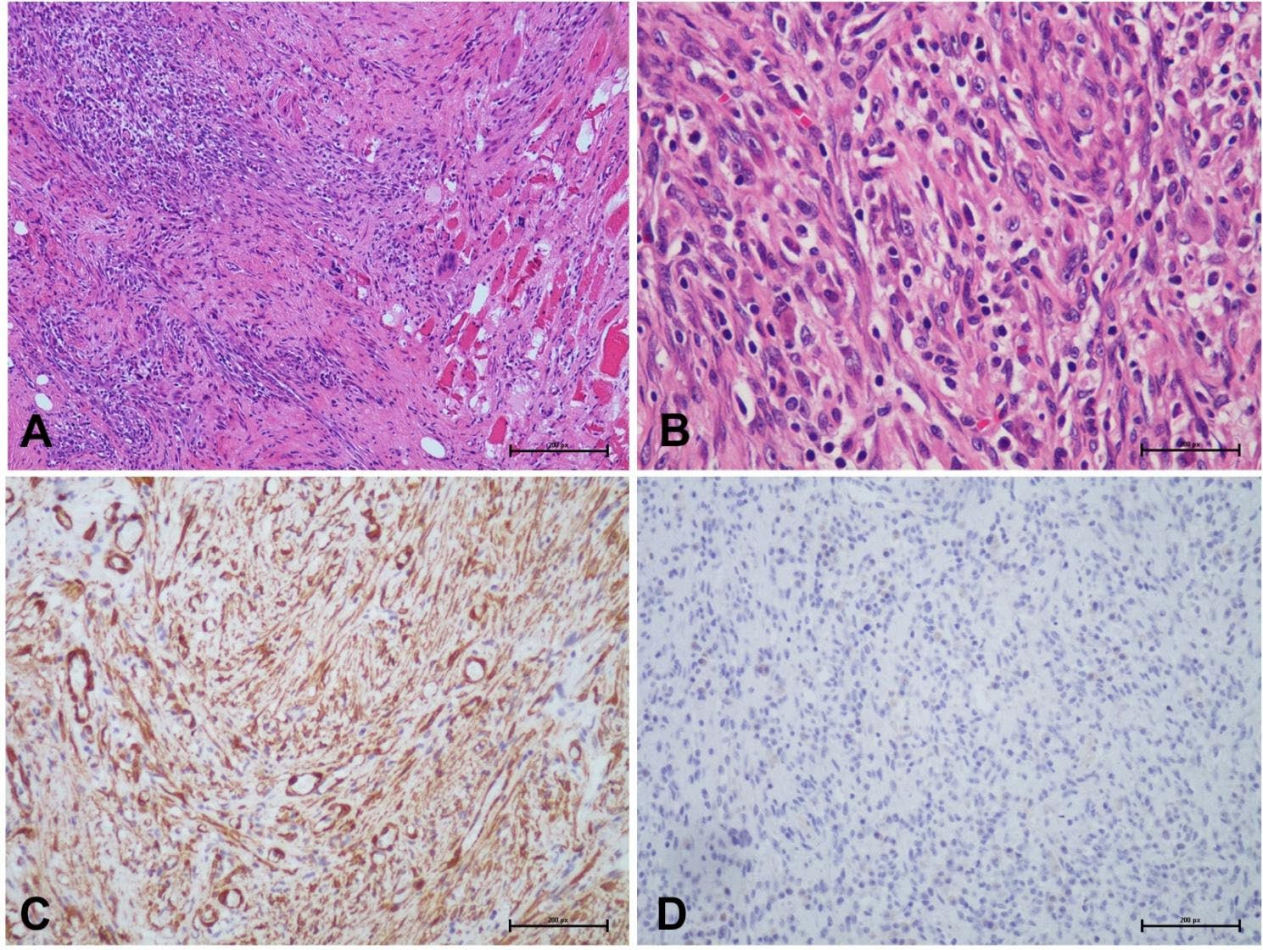
(**A**) The tumor showed spindle cell proliferation with inflammatory infiltration. (**B**) The spindle cells showed a fascicular growth pattern and the infiltration background of plasma cells, neutrophils, and eosinophils. (**C**) SMA immunohistochemical stain shows diffuse positivity of spindle cells. (**D**) The immunohistochemical stain for ALK (1A4) is negative

Macroscopically, the lesions exhibited a white to tan fleshy cut surface. Microscopic examination at low power revealed the dense spindle cells proliferation. The tumor cells were arranged in fascicular or haphazard patterns with irregular borders from surrounding skeletal muscle, accompanied by admixtures of inflammatory infiltrate with an abundance of plasma cells and lymphocytes in a hyalinized or myxoid background. At high-power view, the tumor cells were spindle-shaped with eosinophilic abundant cytoplasm, occasionally lightly vacuolated cytoplasm, and slightly pleomorphism. Nuclei were oval with smooth nuclear contours, pale, dispersed chromatin, and nucleoli were prominent. (Fig. 2A and B) Mitoses were 2/10HPF, and no atypical mitoses were demonstrated. No necrosis was found.

**Fig. 3:**
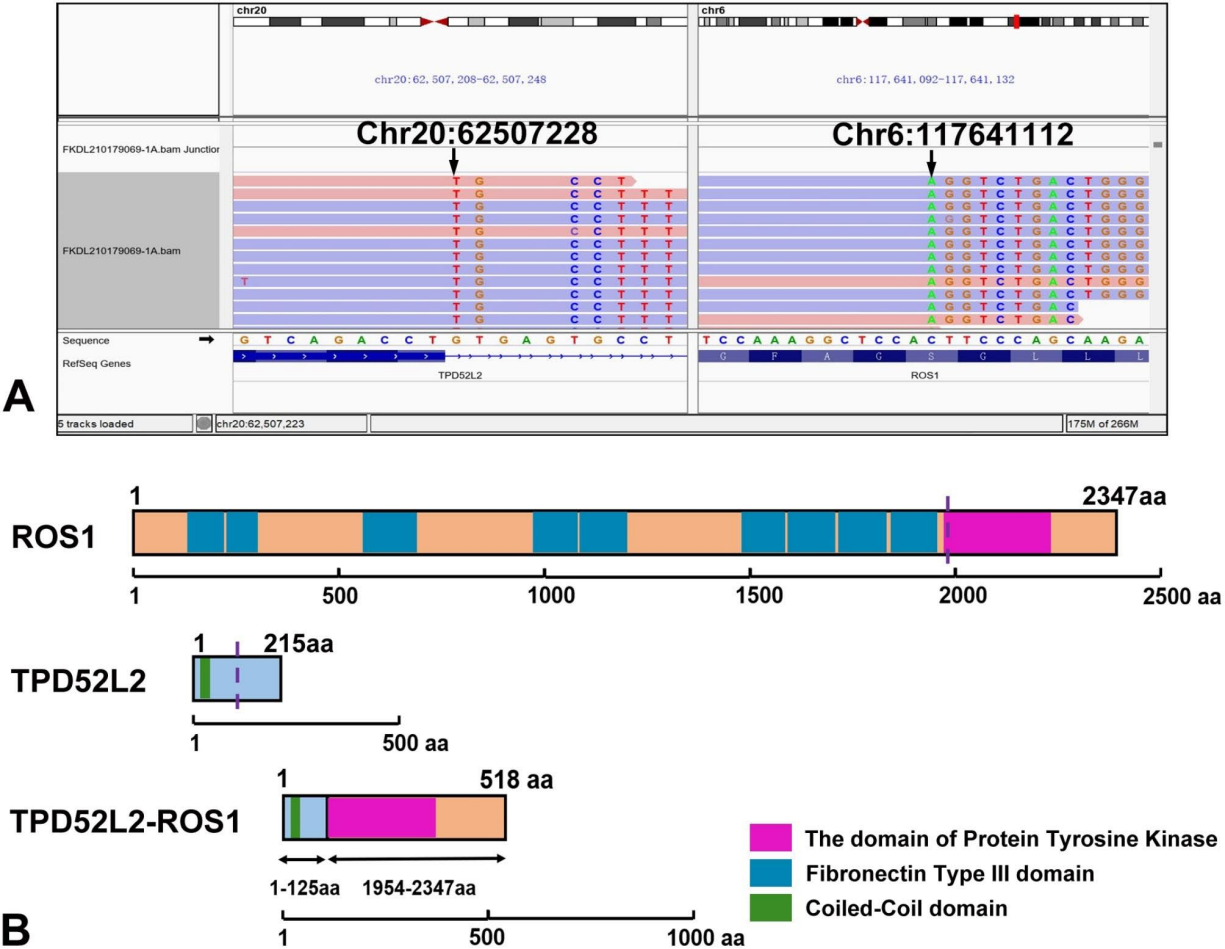
(**A**) Integrated Genomics Viewer snapshot showing paired-end sequencing data demonstrating a somatic intra-chromosomal TPD52L2-ROS1 rearrangement. The arrow points to the breakpoint of both genes. (**B**) The functional domains of the encoded TPD52L2 protein (2347 aa) and ROS1 protein (215 aa). The dotted lines indicate the breakpoints. Schematic representation of the novel TPD52L2-ROS1 chimeric protein (518 aa)

Immunohistochemical staining was diffusely positive for SMA and p16. The staining of MDM2 was moderately positive. The tumor cells did not express pan-cytokeratin, desmin, MyoD1, Myogenin, Melan-A, HMB45, EMA, S-100, SOX-10, IgG4, and pan-TRK. ALK staining showed negative results using two different clones (1A4 and D5F3). (Fig. 2C and D) The Ki-67 index was about 10% in the hot spots.

Given the expression of MDM2 immunohistochemically, fluorescence chromosome in situ hybridization (FISH) for MDM2 was performed, but no copy number variation of MDM2 gene was detected. Moreover, the FISH assay validated that ALK rearrangement was absent.

**Fig. 4:**
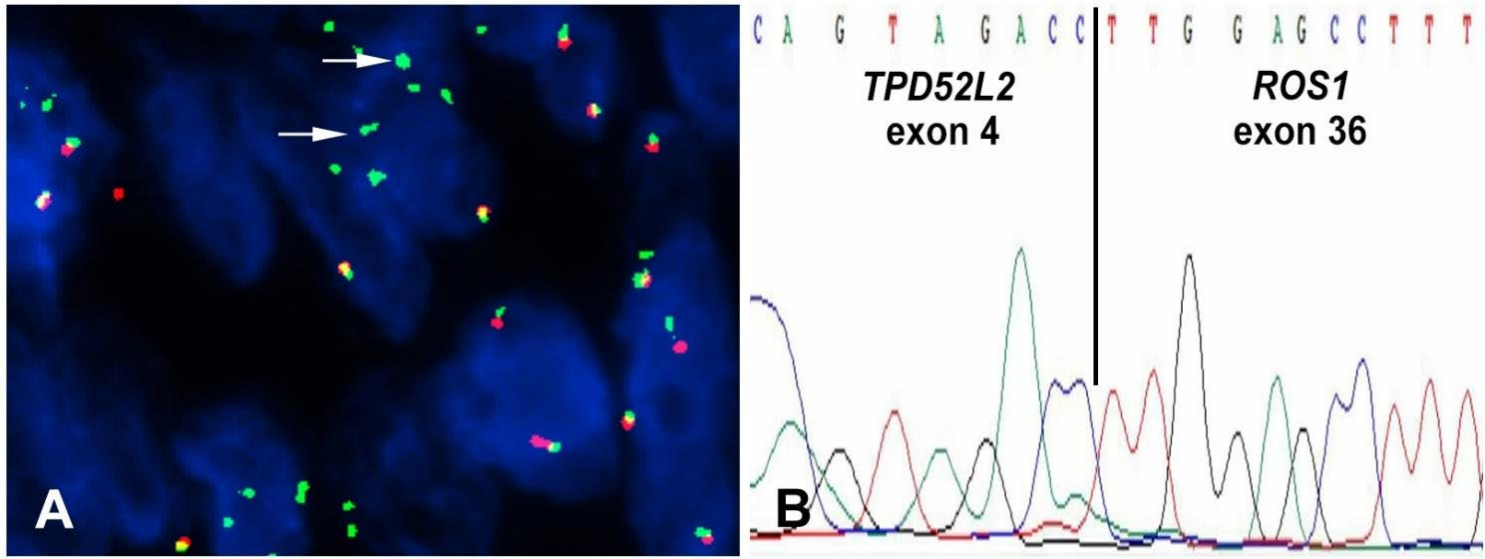
(**A**) *ROS1* (Green) break-apart fluorescence in situ hybridization shows the loss of the 5’ signal (Red) in several cells, indicating positive *ROS1*-related fusion (arrow). (**B**) Sanger sequencing demonstrates that exon 4 of *TPD52L2* is fused with exon 36 of *ROS1*.

Gene fusion analysis was conducted using the targeted Illumina TruSight RNA Fusion Panel which included 507 genes. Sequence reads were aligned to the following genome build: Homo sapiens (human) genome assembly GRCh37 (hg19) from Genome Reference Consortium. A fusion variant between *TPD52L2* 3’ end of exon 1–4 (NM_199360) and *ROS1* 5’ end of exon 36–43 (NM_002944) was revealed. (Fig. 3) The result was validated by the Sanger sequencing. Furthermore, *ROS1* rearrangement was also identified by FISH. ROS1 immunohistochemical staining was negative. (Fig. 4)

In the presence of this *ROS1* mutation, the patient started anlotinib (receptor tyrosine kinases inhibitor, 12 mg orally, daily) as a treatment after the detection. A significant dimensional reduction of the mass was observed (108 mm×86.2 mm, pre-treatment the size was 147 × 116.8 mm) one month later. (Fig. 1B) The movement of the patient’s arm was freer than pretreatment with the medicine, and the swelling was alleviated. Given the good clinical response, the patient did not receive chemotherapy. After two-month treatment with anlotinib, the tumor regression had stagnated, and then crizotinib was used for treatment (250 mg orally, daily) instead of anlotinib. Arm pain and other clinical symptoms were alleviated after one week of treatment. No evidence of progression is observed, and the patient has been in a stable condition. The follow-up is 9 months.

Based on the morphological, immunohistochemical, molecular findings and the good response to receptor tyrosine kinases inhibitors, the final diagnosis of the inflammatory myofibroblastic tumor with a previously undescribed *ROS1* fusion is made, although ALK rearrangement is negative.

## Discussion

Inflammatory myofibroblastic tumors (IMTs) are rare soft tissue neoplasms that usually occur in the lung, abdomen, and pelvis, and can involve any location including extremities. IMTs affect primarily children and adolescents, although they may arise in the eighth decade of life [9].

Histologically, IMT is typically composed of myofibroblastic spindle cells in an inflamed stroma of plasma cells, lymphocytes, and eosinophils [8]. Three basic patterns have been defined, which are often seen in combination within the same tumor: a myxoid/vascular pattern, a compact spindle cell pattern, and a fibromatosis-like pattern [10]. In the present case, the compact spindle cell pattern was predominant with areas of plate-like collagen and inflammatory infiltration, therefore, IMT should be considered.

Based on typical morphological features, immunohistochemical positive reactivity for ALK is an important diagnostic clue for IMTs. The marker is relatively specific for IMT among the series of fibroblastic-myofibroblastic tumors and other mesenchymal mimics of IMT, while smooth muscle markers (including Desmin, h-caldesmon, and SMA), CD34, and MDM2 are expressed variably, which are not distinct for IMTs [7, 11, 12].

*ALK* gene rearrangement was identified in approximately 50% of IMTs, and the recognition of *ALK* gene rearrangements could be helpful to differentiate this entity from other mimics. In some cases, ALK IHC staining was negative, as well as in the present case, however, the diagnosis of IMT could not be excluded. Furthermore, the FISH or NGS assay should be performed for *ALK* gene. Recent advances have revealed that IMTs harbored other multiple potentially actionable kinase fusions, such as *PDGFRB*, *RET*, *ROS1*, and *NTRK3*, which had expanded the molecular spectrum of IMTs [4, 6–8, 11, 13, 14]. The recognition of such gene fusions was helpful to identify the ALK-negative IMT.

*ROS1* rearrangement has been found not only in the epithelial tumors, such as non-small cell lung cancer (NSCLC), ovarian cancer, gastric adenocarcinoma, and colorectal cancer, but also in some mesenchymal tumors, such as IMT, angiosarcoma, and leiomyosarcoma [14–17]. In IMT, *ROS1* fusion was reported in about 10% of cases to date, and fusions of *ROS1* with *SLC12A2*, *YWHAE1*, *TFG*, and *FN1* have been described previously [4–6, 8, 11, 18]. According to the literatures, the IMTs with ROS1 fusion usually occurred in children and adolescents. The average age was 17 years old. The IMTs with ROS1 fusion was reported in some unusual anatomic sites, such as liver, pharynx, buttock, esophagus, pelvis, and scapula, except for lung and abdominal. The involved breakpoint of *ROS1* fusions in IMTs invariably occurs at the exons 32–37, which encodes the variable region of ROS1 kinase protein in the transmembrane region of the plasma membrane. The DNA fragment involving the *ROS1* 3’ region (that encodes the intracellular kinase domain) fused to partner genes leading to promote tumorigenicity [4, 6, 11, 16, 18–23].

In this case, a novel *ROS1* fusion, *TPD52L2-ROS1*, was revealed by NGS, which has not been reported in IMT and other tumors. *TPD52L2* gene belongs to the D52 protein family genes (including *TPD52*, *TPD52L*1, and *TPD52L2*). The rearrangement fusion of this family gene was rare. To date, there was no report about *TPD52L2* gene fusion in the tumor, except for our case. Meanwhile, *TPD52L1-ROS1* fusion was reported in NSCLC, and the breakpoint of *ROS1* and *TPD52L1* was located in intron 32 and intron 3, respectively [24].

The TPD52L2 protein, a cell cycle-regulated protein, retains its coiled-coil domain (amino acids 38–82) that is necessary for homo- and heterodimerization [25]. The fusion gene could encode TPD52L2-ROS1 chimeric protein which contains the ROS1 kinase domain in the C-terminal and the part of TPD52L2 protein (amino acids 1–125) in the N-terminal. According to the previous reports, all known ROS1 chimeric partners contain dimerization domains that turn the fusion kinase into homodimers leading to a constitutively activated state. Notably, an optimistic clinical response was observed after using the receptor tyrosine kinases inhibitor (Anlotinib and Crizotinib) in the present case. We inferred that the TPD52L2-ROS1 chimeric protein could involve in the kinase activation and tumor development.

The differential diagnosis of IMT depends on the site, age, morphologic characteristics, and molecular features. ALK IHC staining could be a useful marker for the diagnosis of this neoplasm. However, the diagnosis of *ALK*-negative spindle cell neoplasm with inflammation could be a significant challenge, especially, when tumors arose in older patients or at unusual anatomic sites, or tumors showed atypical spindle cells. In the present case, the spectrum of differential diagnosis is broad and includes benign and malignant neoplasm, such as nodular fasciitis, low-grade follicular dendritic cell sarcoma, low-grade myofibroblastic sarcoma, synovial sarcoma, and some other high-grade sarcomas.

When IMT is composed of relatively uniform, plump myofibroblastic spindle cells and infiltrative border, especially in young adults’ extremities, it could be misdiagnosed as nodular fasciitis. Nodular fasciitis shows a cell culture-like growth pattern and is often bordered by thin-walled capillaries that resemble granulation tissue. Moreover, these lesions often present with rapid clinical growth. The immunohistochemical staining profile of nodular fasciitis is non-specific. However, the diagnosis of nodular fasciitis can be excluded when ALK staining is positive. USP6 rearrangements in nodular fasciitis play a key role in the differential diagnostic process. Low-grade follicular dendritic cell sarcoma is characteristic of the admixture of cellular fascicles of bland spindle cells and chronic inflammation, and the nuclear of tumor cells harbor fine chromatin. However, the positive expression of follicular dendritic cell markers, including CD21 and CD35, could be found in follicular dendritic cell sarcoma, instead of IMT. In addition, low-grade myofibroblastic sarcoma (LGMS) could be misdiagnosed as IMT. LGMS usually affects older adults and has a predilection for the head and neck region. LGMS tends to be a more uniform pattern with higher cellularity, more prominent nuclear atypia, more frequent mitoses, and a more widely infiltrative growth pattern than IMT. Immunohistochemical stains for myofibroblast-associated markers are not particularly useful in making the distinction between IMT and LGMS. ALK expression is not found in LGMS, which could be used as an adjunct to differential diagnosis. Synovial sarcoma should be considered in the differential diagnosis, and both are predominant in young adults, especially, when the tumor is characterized by monophasic spindle cells and inflammatory infiltration. Synovial sarcoma is positive for CD99, TLE1, focally AE1/AE3, and EMA, and has a characteristic SS18-SSX gene fusion. When the arrangement of tumor cells is compact and characterized by nuclear pleomorphism, some high-grade sarcomas should also be distinguished from IMT. Dedifferentiated liposarcoma could be a pitfall, given a great proportion of IMT shows MDM2 positive. Nevertheless, dedifferentiated liposarcoma usually occurs in the retroperitoneum in an older age group, and the area of well-differentiated liposarcoma components could be observed.

In summary, we report a rare case of ALK-negative IMT harboring a previously undescribed *TPD52L2-ROS1* fusion which expands our understanding of the spectrum of gene fusions in this type of fibroblastic/myofibroblastic neoplasm. NGS has been an essential approach to reveal novel gene fusions in soft tissue tumors. In addition to IMT, receptor tyrosine kinase gene fusion has been reported in a variety of soft tissue neoplasms, such as S100/CD34-positive spindle cell mesenchymal neoplasm. Such advances expand our understanding of molecular pathogenesis to these types of mesenchymal tumors and might avoid diagnostic pitfalls, on the other hand, give the patients promising therapeutic strategies.

## Data Availability

The data that support the findings of this study are available from the corresponding author upon reasonable request.
